# Characterization and Properties of Activated Carbon Prepared from Tamarind Seeds by KOH Activation for Fe(III) Adsorption from Aqueous Solution

**DOI:** 10.1155/2015/415961

**Published:** 2015-11-25

**Authors:** Sumrit Mopoung, Phansiri Moonsri, Wanwimon Palas, Sataporn Khumpai

**Affiliations:** ^1^Department of Chemistry, Faculty of Science, Naresuan University, Phitsanulok, Thailand; ^2^Naresuan University Secondary Demonstration School, Naresuan University, Phitsanulok, Thailand

## Abstract

This research studies the characterization of activated carbon from tamarind seed with KOH activation. The effects of 0.5 : 1–1.5 : 1 KOH : tamarind seed charcoal ratios and 500–700°C activation temperatures were studied. FTIR, SEM-EDS, XRD, and BET were used to characterize tamarind seed and the activated carbon prepared from them. Proximate analysis, percent yield, iodine number, methylene blue number, and preliminary test of Fe(III) adsorption were also studied. Fe(III) adsorption was carried out by 30 mL column with 5–20 ppm Fe(III) initial concentrations. The percent yield of activated carbon prepared from tamarind seed with KOH activation decreased with increasing activation temperature and impregnation ratios, which were in the range from 54.09 to 82.03 wt%. The surface functional groups of activated carbon are O–H, C=O, C–O, –CO_3_, C–H, and Si–H. The XRD result showed high crystallinity coming from a potassium compound in the activated carbon. The main elements found in the activated carbon by EDS are C, O, Si, and K. The results of iodine and methylene blue adsorption indicate that the pore size of the activated carbon is mostly in the range of mesopore and macropore. The average BET pore size and BET surface area of activated carbon are 67.9764 Å and 2.7167 m^2^/g, respectively. Finally, the tamarind seed based activated carbon produced with 500°C activation temperature and 1.0 : 1 KOH : tamarind seed charcoal ratio was used for Fe(III) adsorption test. It was shown that Fe(III) was adsorbed in alkaline conditions and adsorption increased with increasing Fe(III) initial concentration from 5 to 20 ppm with capacity adsorption of 0.0069–0.019 mg/g.

## 1. Introduction

Tamarind (*Tamarindus indica* L.) was planted on a large scale in India, Thailand, Indonesia, Myanmar, and the Philippines. Tamarind fruit consists of pulp and hard-coated seeds. The seeds are 30–40% of fruit with a large quantity as agrobyproduct [1]. Tamarind seed powder has been used as a biosorbent for removal of Cr(VI) from simulated industrial wastewater [2]. Tamarind seed has also been used as a raw material for granular activated carbon prepared by microwave induced chemical activation for the adsorptive treatment of semiaerobic landfill leachate [3]. In addition, tamarind seed was activated by H_2_SO_4_ at 150°C. The activated carbon product could adsorb Cr(VI) up to 29.7 mg/g at pH 1–3 [4].

Pollution of natural water resources by iron is one of the most important problems that face and threaten the world. Fe contaminants are produced from liquid wastes discharged from a number of industries [5]. Water with high iron content can be very objectionable in taste, odour, or appearance. With severe iron poisoning, much of the damage to the gastrointestinal tract and liver may be the result of highly localized iron concentration and free radical production leading to hepatotoxicity through lipid peroxidation and the destruction of the hepatic mitochondria. With this result, the liver becomes cirrhotic. Hepatoma, the primary cancer of the liver, has become the most common cause of death among patients with hemochromatosis [6]. Activated carbon has been proved to be an excellent adsorbent for removing organic or inorganic pollutants [7]. It could be produced from agricultural byproducts with low cost and abundance [8].

In this research, tamarind seed was used as precursor for the production of activated carbon using KOH activation. The effects of KOH : tamarind seed ratios and activation temperature were studied. The physical and chemical properties were characterized. The adsorption of iodine and methylene blue was also determined. Finally, the activated carbon prepared in this study was used in preliminary test for Fe(III) adsorption from aqueous solution.

## 2. Materials and Methods 

### 2.1. Preparation of Material

Tamarind seed (sweet Thai tamarind) was obtained from Nakhon Thai district, Phitsanulok province, Thailand. It was washed and oven dried (SL 1375 SHEL LAB 1350 FX) at 105°C for 3 h. Proximate analysis was used to determine the ash content [9], volatile matter [10], fixed carbon [11], and moisture content [12].

### 2.2. Preparation of Charcoal

Accurately weighed samples of dried tamarind seed (weighed with analytical balance, Sartorius Basic) were carbonized in a closed crucible (size 105/73 and 102/70) at 500°C in furnace (Fisher Scientific Isotemp Muffle Furnace) for 1 h. The charcoal product was ground and sieved to 2 mm size. The percent yield of tamarind seed based charcoal is 40.14%.

### 2.3. Preparation of Activated Carbon

The tamarind seed charcoal was mixed with KOH (CARLO ERBA Reagent) using KOH to tamarind seed charcoal ratios of 0.5 : 1, 1.0 : 1, and 1.5 : 1 (wt/wt). The mixtures were activated at 500°C, 600°C, and 700°C in furnace. Fourier transform infrared spectrometer (Spectrum GX, PerkinElmer) in the range of 4000–400 cm^−1^ was used for characterization of functional groups on surface of the all samples. The samples were prepared as pellets using KBr (IR grade) [13]. An X-ray powder diffractometer with a Cu tube anode (PW 3040/60, X'Pert Pro MPD) was used to record the X-ray patterns of samples. Scanning electron microscopy (PHILIPS LEO 1455 VP) was used to visualize the surface morphology of the carbonized and activated products. The samples were coated with gold by a gold sputtering device for a clear vision of the surface morphology. Elemental composition of these samples was also determined using scanning electron microscopy equipped with energy dispersive spectrometer (EDS). The EDS spectra showing elemental composition were obtained by scanning through the surfaces of the samples. The surface distributions were collected from SEM pictures using different magnifications. Textural characteristics of only activated carbon prepared using the 1.0 : 1 KOH : charcoal ratio with 500°C activation were determined by N_2_ adsorption at −196°C on Brunauer-Emmett-Teller surface area analyzer (Micromeritics TriStar II). The samples were degassed at 250°C for 12 h under vacuum before the measurements. The specific surface areas were estimated by the multipoint Brunauer-Emmett-Teller (BET) equation. The iodine (0.1 N, CARLO ERBA Reagent) and methylene blue (100 ppm, AR UNILAB) numbers were also determined for all activated carbons.

### 2.4. Fe Adsorption

The activated carbon prepared using 1.0 : 1 KOH to charcoal mixture with activation at 500°C was used for Fe adsorption experiment. Accurately weighed samples of activated carbon were filled into 30 mL columns. Solutions of FeCl_3_ (AR UNILAB) at initial concentrations of 5, 10, and 20 ppm (pH 7.96 ± 0.33) were passed through the column. The eluted solutions were collected for Fe(III) concentration determination by AAS Varian SpectrAA 220 (Australia). Fe(III) adsorptions were calculated in mg Fe/g activated carbon. The pH of Fe(III) solution and leachate were measured by pH meter (HORIBA F-21, Japan). The pH of activated carbon (1 : 1 weight/volume of activated carbon to H_2_O) was also measured by method of Bansode et al. [14].

## 3. Results and Discussion

### 3.1. Proximate Analysis of Tamarind Seed and Percent Yield of Activated Carbon

The proximate composition of tamarind seed is 1.14 ± 0.07 wt% moisture, 1.65 ± 0.04 wt% ash, 67.64 ± 1.30 wt% volatile matter, and 28.89 ± 0.45 wt% fixed carbon, which makes it suitable precursor for obtaining activated carbon [15].

**Figure 1:**
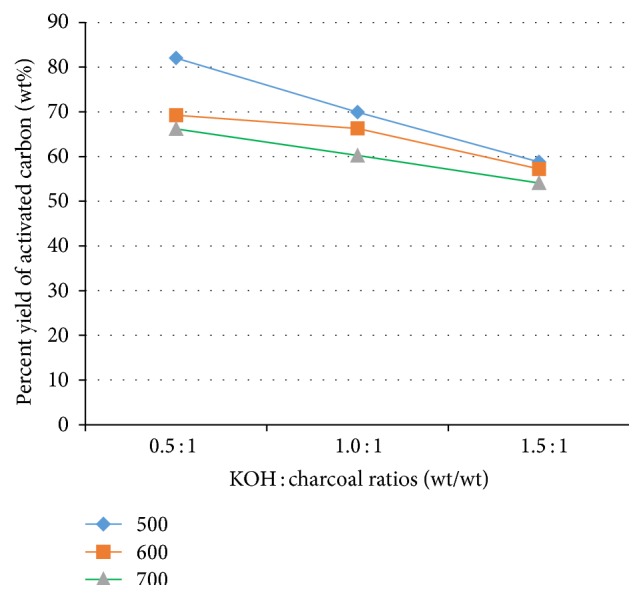
Percent yield of tamarind seed based activated carbon as a function of KOH : tamarind seed charcoal ratio and activation temperature.

The percent yield of tamarind seed based activated carbon decreases as activation temperature increases from 500 to 700°C (Figure 1). This could be attributed to the higher reaction rate of carbon and KOH to release more volatile components with concomitant improvement in the textural characteristics [16] and carbon burn-off [17].

### 3.2. Adsorption of Iodine

Determining the iodine number is one of the methods to determine the adsorption capacity of activated carbons. It is a measure of the micropore (0–20 Å) content of the activated carbon by adsorption of iodine from solution. The typical range is 500–1200 mg/g, which is equivalent to surface area of carbon between 900 and 1100 m^2^/g [17].

**Figure 2:**
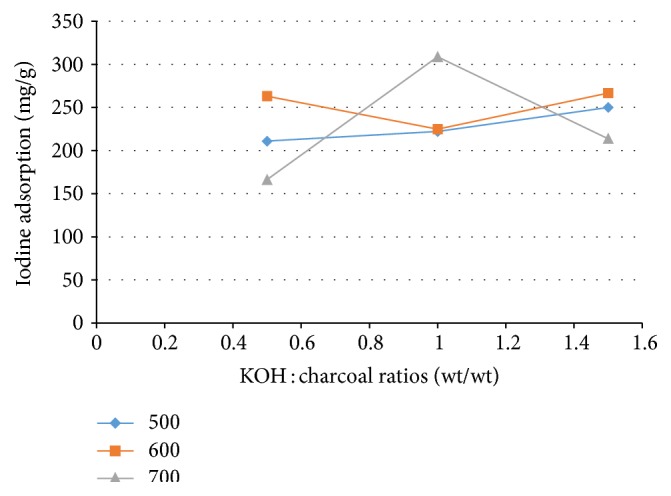
Iodine adsorption of tamarind seed based activated carbon as a function of KOH : tamarind seed charcoal ratio and activation temperature.

It can be seen from Figure 2 that iodine adsorption of activated carbon prepared with activation at 500°C slightly increases with increasing impregnation ratios. It was shown that micropore content on surface of activated carbon is slightly increased with increasing impregnation ratios. This is attributed to more extensive reaction between KOH and surface carbon [18], leading to increased release of CO_2_ and CO gases and creating micropores inside of the mesopores [19]. The iodine adsorption of activated carbon prepared with activation temperature of 600°C and 1.0 : 1 impregnation ratio is lower than that of activated carbon prepared with impregnation ratios of 0.5 : 1 and 1.5 : 1. This may be attributed to more extensive reaction of KOH and surface carbon at 0.5 : 1 impregnation ratio [18] and more excessive carbon burn-off at 1.5 : 1 impregnation ratio [17]. However, the iodine adsorption of activated carbon prepared with activation temperature of 600°C is higher than that of activated carbon prepared with activation temperature of 500°C for all impregnation ratios. This is due to more extensive volatile matter degradation and increased reaction of KOH and surface carbon at higher activation temperature [20]. The iodine adsorption at the activation temperature of 700°C was highest for activated carbon prepared using 1.0 : 1 impregnation ratio. The iodine adsorption of activated carbon prepared with 1.5 : 1 impregnation ratio was higher than that of activated carbon prepared with 0.5 : 1 impregnation ratio. It was shown that more micropores are created at impregnation ratios <1.0 : 1, but the content of micropores decreases at impregnation ratios ≥1.0 : 1 due to more excess carbon burn-off, collapse of pore walls [18], or expansion of micropores to mesopores [19]. Furthermore, the reaction between carbon and KOH may damage the micropores on the carbon surface because the concentration of KOH solution is too high [21].

### 3.3. Adsorption of Methylene Blue

Activated carbon is amphoteric material, which can be positively or negatively charged depending on the solution pH. Attraction between activated carbon and anionic or cationic guest materials is mainly related to the surface characteristics. More negatively charged surfaces are obtained at higher pH values and this favors the uptake of more cationic groups due to decreased electrostatic repulsion between cations and the surface of activated carbon and vice versa [22]. Methylene blue (C_16_H_18_N_3_SCl) is a cationic dye with the estimated dimensions of 1.43 nm × 0.61 nm × 0.4 nm and its adsorption by activated carbon is very susceptible to solution pH. The adsorption of methylene blue involves the electrostatic interaction of methylene blue cations with negatively charged carbon surface functional groups [22].

**Figure 3:**
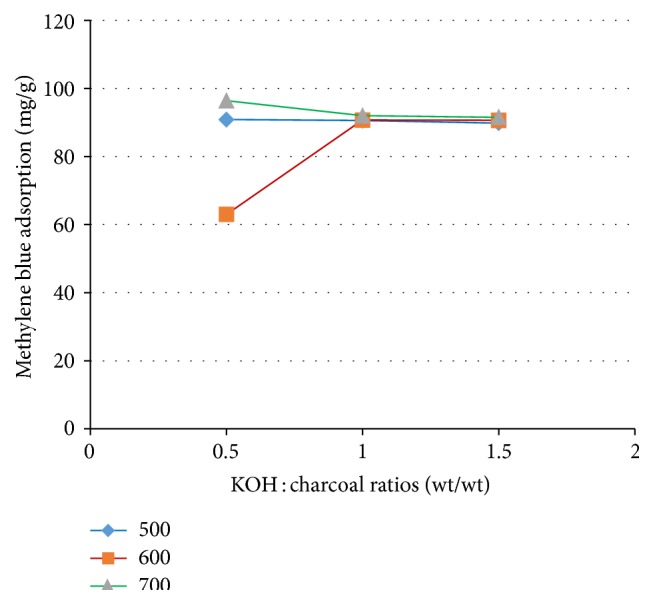
Methylene blue adsorption of tamarind seed based activated carbon as a function of KOH : tamarind seed charcoal ratio and activation temperature.

Figure 3 shows the methylene blue adsorption on tamarind seed based activated carbon. It was shown that the methylene blue adsorption capacity of tamarind seed based activated carbon is very high. This revealed the fact that the pore size of activated carbon is mainly in the mesopore regime and has negative surface charge, which corresponds to resorcinol-formaldehyde based activated carbon [19] and corncob based activated carbon [23], with KOH activation. Tamarind seed has pores with size mainly in the mesopore range [3]. As a result, tamarind seed based activated carbon is mainly mesoporous and has high methylene blue adsorption capacity. Another effect is the surface charge of activated carbon, which is negative at high pH [22]. The methylene blue adsorption on tamarind seed based activated carbon activated at 500°C is relatively constant for all impregnation ratios of KOH : tamarind seed. This could be explained by relatively small change in the mesopore portion of initial tamarind seed during activation at 500°C. For activated carbon prepared at 600°C, the methylene blue adsorption increases with impregnation ratio rising from 0.5 : 1 to 1.0 : 1 and then remains constant. At impregnation ratio of 0.5 : 1 used for the preparation of activated carbon at 600°C, the methylene blue adsorption is lower than that of activated carbons prepared at 500°C and 700°C. It may be attributed to new micropores being created inside of the mesopores. This is the result of a higher coverage activated carbon surface with potassium compounds, which will inhibit the physical surface [23]. Another reason might be the decomposition of negatively charged surface groups with more material being released by KOH activation at high temperature, especially OH group of KOH [16]. At increasing impregnation ratios more volatile matter is released and more carbon burn-off takes place [17] leading to more mesopores or macropores. For carbon activated at 700°C, there is a slight downward trend from ratio 0.5 : 1 to 1.0 : 1 and then methylene blue adsorption remains constant. However, the methylene blue adsorption is nearly equal for reagent ratios 1.0 : 1 and 1.5 : 1 at all of the activation temperatures. This can be explained by the mesopore remaining unchanged with increasing burn-off [24]. Additionally, the charcoal starting materials were soaked in a large amount of KOH and a thin film of KOH should be coated on their surface and the interior of charcoals should be covered with KOH completely. During the activation in an inert atmosphere surface pyrolysis does not occur on the surface of chars, causing the surface structure to remain [25]. Finally, the strongly basic concentrated KOH solution might also destroy the pore structure of carbon [21]. These results are in accordance with the results of BET, where the average BET pore size of activated carbon is 67.9764 Å.

### 3.4. SEM-EDS

**Figure 4:**
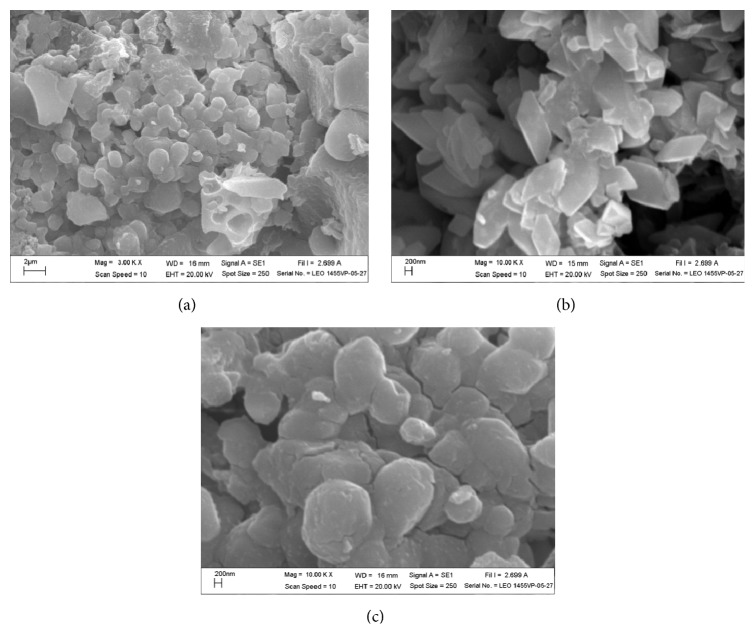
Scanning electron micrographs (a–c) and elemental composition determined by EDS (Table 1) of tamarind seed based activated carbon prepared with 1.0 : 1 impregnation ratio and activated at 500°C.

SEM images of tamarind seed based activated carbon with 1.0 : 1 impregnation ratio at an activation temperature of 500°C are shown in Figure 4. It can be seen from the micrographs that the external surface of the activated carbon particles has cracks, crevices (Figures 4(a) and 4(c)), and some crystals of various sizes in large holes. The crystals in the macropores (Figure 4(b)) are most likely the potassium compounds as is hinted at by the results of the EDS. The EDS showed (Table 1) high content of potassium in the activated carbon. This is due to activation in the presence of KOH, where K_2_CO_3_ and other related compounds form during the pyrolysis process [18]. This result is also in good accordance with the results of BET because there is large amount of potassium compounds covering the surface of the activated carbon, leading to relatively low BET surface area of only 2.7167 m^2^/g.

Elemental analysis of the residue found the presence of C, O, Si, and K (Table 1). Furthermore, it was found that carbon content was only 22.96 wt%. This result is attributed to the reaction between KOH and C, which is considered the main reaction [23]. It is expected that large amount of carbon decomposed by reaction with potassium hydroxide [16]. Therefore, the activated carbons obtained by KOH activation have higher potassium and oxygen contents. The content of Si in the activated carbon roughly corresponds to its content in the original tamarind seed.

### 3.5. FTIR Spectra

**Figure 5:**
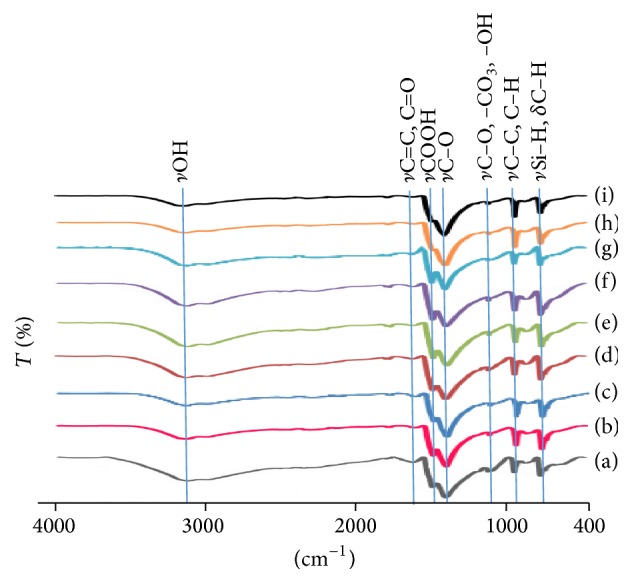
FTIR transmission spectra of (a) activated carbon prepared with 0.5 : 1 impregnation ratio and activated at 500°C, (b) activated carbon prepared with 1.0 : 1 impregnation ratio and activated at 500°C, (c) activated carbon prepared with 1.5 : 1 impregnation ratio and activated at 500°C, (d) activated carbon prepared with 0.5 : 1 impregnation ratio and activated at 600°C, (e) activated carbon prepared with 1.0 : 1 impregnation ratio and activated at 600°C, (f) activated carbon prepared with 1.5 : 1 impregnation ratio and activated at 600°C, (g) activated carbon prepared with 0.5 : 1 impregnation ratio and activated at 700°C, (h) activated carbon prepared with 1.0 : 1 impregnation ratio and activated at 700°C, and (i) activated carbon prepared with 1.5 : 1 impregnation ratio and activated at 700°C.

Figure 5 shows the FTIR spectra of tamarind seed based activated carbon prepared with KOH to tamarind seed charcoal ratios of 0.5–1.5 and activated at 500–700°C. It can be seen that all of spectra are similar. There are a broad band at about 3200 cm^−1^, very weak peak at 1600 cm^−1^, strong peak at 1400 cm^−1^, shoulder peak at 1500 cm^−1^, very weak peak at 1150 cm^−1^, and weak peaks at 900 cm^−1^ and 700 cm^−1^. The broad band at 3200 cm^−1^ associated with –OH stretching vibration of hydroxyl functional groups. The very weak peak at 1600 cm^−1^ corresponds to C=C stretching of the aromatic rings [16]. These could form by decomposition of C–H bonds to form a more stable aromatic C=C bonds at the higher activation temperatures [26]. This feature may also be due to C=O groups conjugated with aromatic rings [27]. This indicates the formation of carbonyl-containing groups formed during the aromatization of tamarind seed [28]. The intensity of this peak gradually decreases with increasing activation temperatures and impregnation ratios. The shoulder peaks at about 1500 cm^−1^ and 1400 cm^−1^ are attributed to carboxyl-carbonate structures [21]. The peak at 1400 cm^−1^ can be attributed to oxygen containing functional groups, for example, C=O and C–O of carboxylic groups [27] or in-plane vibration of O–H of carboxylic group [29]. The very weak peak at about 1150 cm^−1^ corresponds to the stretching vibration of C–O group in alcohol, phenol, ether, or ester [17]. It could also be attributed to carboxylic groups [29], including –CO_3_ group [18] and the phenolic –OH group [21]. The peak at 960 cm^−1^ is associated with stretching vibration of C–C or C–H groups [30] or C=O group [31]. The peak at about 700 cm^−1^ may be due to Si–H stretching vibration [30] or polycyclic and C–H bending vibration of benzene rings [28]. These functional groups can have negative or positive charge depending on the solution pH [22].

As compared to tamarind seed char which had been prepared by Munusamy et al. [32], it showed the peaks of O–H group (3334 cm^−1^), C–H group (2879 cm^−1^), C=C group (1548 cm^−1^), and C–C group (1165 cm^−1^). It was shown that NaOH activation resulted in increased functional groups, except CH group which disappeared after activation.

### 3.6. XRD Diffractograms

**Figure 6:**
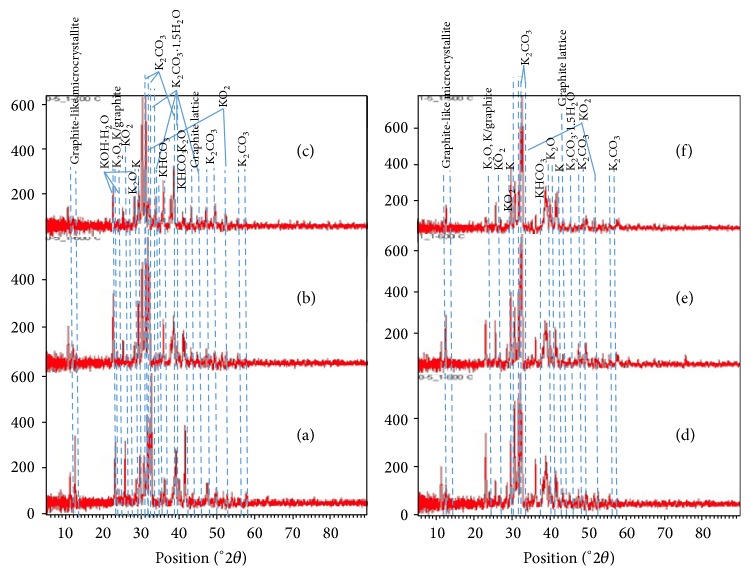
XRD diffractograms of (a) activated carbon prepared with 0.5 : 1 impregnation ratio and activated at 500°C, (b) activated carbon prepared with 0.5 : 1 impregnation ratio and 600°C activation temperature, (c) activated carbon with 0.5 : 1 impregnated ratio and 700°C activation temperature, (d) activated carbon with 0.5 : 1 impregnated ratio and 600°C activation temperature, (e) activated carbon with 1.0 : 1 impregnated ratio and 600°C activation temperature, and (f) activated carbon with 1.5 : 1 impregnated ratio and 600°C activation temperature.

The result of XRD diffractograms (Figure 6) revealed that the activated carbons may contain potassium compounds with high crystallinity after activation with KOH. When compared to tamarind seed char [32], it showed only amorphous carbon (two broad peaks at around 2*θ* = 24° and 42°). This finding is similar to the petroleum coke based activated carbon prepared with K_2_CO_3_ activation [33] and* Enteromorpha prolifera* based activated carbon prepared with KOH activation [29]. The reaction of KOH and surface carbon (C_f_) occurring during activation process is as follows [27]:

(1) 4KOH+Cf⟶K2CO3+K2O+2H2

(2) K2CO3+2Cf⟶2K+3CO

(3) K2O+C⟶C–O–K+K

(4) C–O–K+H2O⟶C–O–H+KOH

The reaction on surface of carbon could be explained by the following equation [23]:

(5) 2KOH⟶K2O+H2O  dehydration

(6) Cf+H2O⟶H2+CO  water-gas  reaction

(7) CO+H2O⟶H2+CO2  water-gas  shift  reaction

(8) K2O+CO2⟶K2CO3  carbonate  formation

The final product of activation in the presence of KOH during the preparation of activated carbon is K_2_CO_3_ (8). K metal was produced during the activation process at > 700°C as shown in following equation [23]:

(9) K2O+H2⟶2K+H2O  reduction  by  hydrogen

(10) K2O+Cf⟶2K+CO  reduction  by  carbon

Reactions (9) and (10) occurred at temperatures above 780°C, which is the boiling point of potassium, leading to high loss of potassium [23]. During KOH activation process a gasification reaction occurred in which KOH was reduced to K metal. K_2_CO_3_, which also formed during the activation, was reduced by carbon to K, K_2_O, CO, and CO_2_ and porous carbon surface was also created at the same time. Since the boiling point of K is 780°C, then potassium metal remains in the activated carbon [18].

Figure 6(a–c) shows the effect of activation temperature (500–700°C) with impregnation ratio of 0.5 : 1 on crystallinity of the activated carbon. The effect of impregnation ratios (0.5 : 1–1.5 : 1) on the crystallinity of activated carbon prepared at activation temperature of 600°C is shown in Figure 6(d–f). These show presence of peaks of many potassium compounds. For example, the peaks at 23°, 27°, and 39.5° correspond to K_2_O, which forms at temperatures above 500°C. The intensity of these peaks increases with increasing activation temperature (Figure 6(a–c)), but their intensity decreases with increasing impregnation ratio for materials prepared at 600°C (Figure 5(d–f)). The peaks at 22.5°, 23.5°, 28°, and 31.5° belong to peaks corresponding to KOH·H_2_O. These peaks are very weak at activation temperatures above 500°C. This shows that C reduced almost all KOH into K_2_CO_3_ and K. The peaks corresponding to K metal appear at 29° and 42°. These peaks appear for materials prepared at 500°C and above. Furthermore, the intensity of these peaks tends to increase as the activation temperature and impregnation ratios increase. The peaks of K_2_CO_3_ and K_2_CO_3_·1.5H_2_O occur at 30.5°, 31.5°, and 38.5° and 32°, 33°, 39.5°, and 46.5°, respectively. The small peaks at 46°, 49°, 56°, and 58° can also be attributed to K_2_CO_3_ [34]. The intensity of these peaks increased with increasing impregnation ratios, which is associated with increased reduction of KOH. However, the intensity of the peaks of K_2_CO_3_·1.5H_2_O decreased as activation temperature increased. This indicates that the extent of dehydration was higher with increasing activation temperature. As a result of this, the peaks corresponding to K_2_CO_3_ increase in intensity as the activation temperature increases. The peaks of KO_2_ and ordered K/graphite occurred at 26° and 31° and at 53° and 23°, respectively. The presence of these species is the result of partial oxidation of potassium graphite intercalate via chemical reduction of C–O–C or C–O–H surface functional groups [35]. The content of KO_2_ decreases as activation temperature increases but increases as impregnation ratios increase. Furthermore, the peaks of ordered K/graphite decrease with increasing activation temperature and decreasing impregnation ratios. This suggests that, after activation, activated carbon had an amorphous structure and the graphite crystallites are destroyed by K intercalation [27]. The peaks at 35°, 37°, and 28° associated with KHCO_3_, which formed from interaction of K_2_CO_3_, H_2_O, and CO_2_ in air during cooling stage [36]. Its content increased with increasing activation temperatures and impregnation. These results are in agreement with the results of FTIR and EDS, which showed presence of –OH and –CO_3_ groups and K, respectively. The low intensity peaks at around 23.5° and 44° correspond to the graphite lattice, which demonstrate that the degree of ordered graphitization of activated carbon decreases at higher activation temperatures and impregnation ratios [37]. Finally, the peaks between 10° and 20° are attributed to the presence of micropores and microcrystallinity in the activated carbon [26], which may be due to graphite-like microcrystalline structure of multilayer stacks [38].

### 3.7. Fe(III) Adsorption

**Figure 7:**
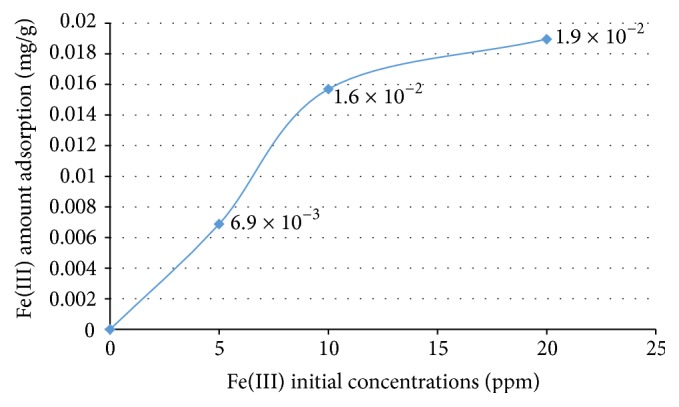
Fe(III) adsorption on tamarind seed based activated carbon as a function of initial Fe(III) concentration.

Fe(III) adsorption capacity of tamarind seed based activated carbon increased (0.0069–0.019 mg/g) with initial 5–10 ppm Fe(III) concentration (Figure 7). This result can be attributed to the higher initial concentration providing a higher driving force to overcome mass transfer resistance. It may also be due to higher interaction between Fe(III) and activated carbon. As the activated carbon offers a finite number of surface binding sites, Fe(III) adsorption showed a saturation trend at higher initial Fe(III) concentration [30]. In this experiment, the pH of Fe(III) solution is 7.96 ± 0.33, which is higher than the pH_ZPC_ (zero point of charge) of tamarind seed based activated carbon (6.00) as reported by Agarwal et al. [2] under similar conditions. At pH > 6, the net surface charge of tamarind seed based activated carbon is negative [2]. Therefore, Fe(III) cations can be adsorbed on the negatively charged surface of activated carbon at solution pH above 6 [39].

## 4. Conclusion

Tamarind seed could be used for charcoal and activated carbon preparation. The yield of charcoal product from tamarind seed prepared by carbonization at 500°C is 40.14 wt%. The percent yield of activated carbon prepared from tamarind seed with KOH activation ranges from 54.09 to 82.03 wt% using impregnation ratios of 0.5 : 1–1.5 : 1 and activation temperatures of 500–700°C. The surface functional groups present on tamarind seed based activated carbon after activation with KOH are O–H, C=O, C–O, –CO_3_, C–H, and Si–H. These functional groups can be negatively or positively charged depending on the solution pH. The XRD results indicate the presence of a range of potassium compounds on the surface of activated carbon, such as K, K_2_CO_3_, K_2_CO_3_·1.5H_2_O, KHCO_3_, KO_2_, and K_2_O. The main elements present in the activated carbon are C, O, Si, and K. The results of iodine and methylene adsorption, SEM, and BET confirmed that the pores of the activated carbon are mostly in range of mesopores to macropores. The average BET pore size of activated carbon is 67.9764 Å. Fe(III) adsorptions capacity of tamarind seed based activated carbon activated at 500°C using KOH to tamarind seed charcoal ratio of 1.0 : 1 is 6.9 × 10^−3^–1.9 × 10^−2^ mg/g, as determined in experiments with initial Fe(III) concentrations of 5–20 ppm at pH 7.96 ± 0.33. The next step, Fe absorption should be further study for find the maximum condition, isotherm and kinetic.

## Figures and Tables

**Table 1:** 

Element	Wt%	At%
C	22.96	37.03
O	34.15	41.35
Si	01.95	01.35
K	40.94	20.28
